# Effective Performance Modifications for a Composite Rocket Propellant via Coagglomerates of Cyclic Nitramines

**DOI:** 10.1021/acsomega.5c01848

**Published:** 2025-05-27

**Authors:** Veerabhadragouda B. Patil, Rafał Lewczuk, Filip Sazeček, Paulina Paziewska, Petr Stojan, Petr Bělina, Svatopluk Zeman

**Affiliations:** ψ Institute of Energetic Materials, Faculty of Chemical Technology, 48252 University of Pardubice, Pardubice CZ-532 10, Czech Republic; Δ Łukasiewicz Research Network - Institute of Industrial Organic Chemistry, Warszawa PL-03-236, Poland; φ OZM Research, Hrochův Týnec CZ-538 62, Czech Republic; ∇ Department of Inorganic Technology, Faculty of Chemical Technology, 48252 University of Pardubice, Pardubice CZ-532 10, Czech Republic

## Abstract

The new combustion modifier cis-1,3,4,6-tetranitrooctahydroimidazo-[4,5-*d*]­imidazole (BCHMX) was tested on an aluminum HMX/HTPB/AP propellant. Monitored were the changes in the propellant characteristics, when the 10, 20, and 30 wt % ammonium perchlorate (AP) were substituted by pure 1,3,5,7-tetranitro-1,3,5.7-tetrazocane (HMX), mechanical mixture HMX/BCHMX (PM), and coagglomerate of HMX/BCHMX (CACs), the latter two in a weight ratio of 8:1. By means of the powder X-ray diffraction and FTIR and Raman spectroscopies, it was confirmed that CACs are a mixture of cocrystal β-HMX/BCHMX with excess β-HMX. The relationships were discovered between the burning rate of the prepared samples and the heat of combustion (*Q*_c_), ignition temperature, specific rate constant of thermal decomposition, impact sensitivity, hardness, and specific impulse. Correspondingly, relationships between *Q*_c_ and ignition temperature, as well as the impact and friction sensitivities, were investigated. It was found that increasing the amount of CACs in the propellant reduces the pressure exponent, while pure HMX and PM have the opposite effect. The significantly positive effect of CACs on the burning rate, especially at 20 wt % substitution of AP by these microcrystals, is another noteworthy finding.

## Graphical abstract

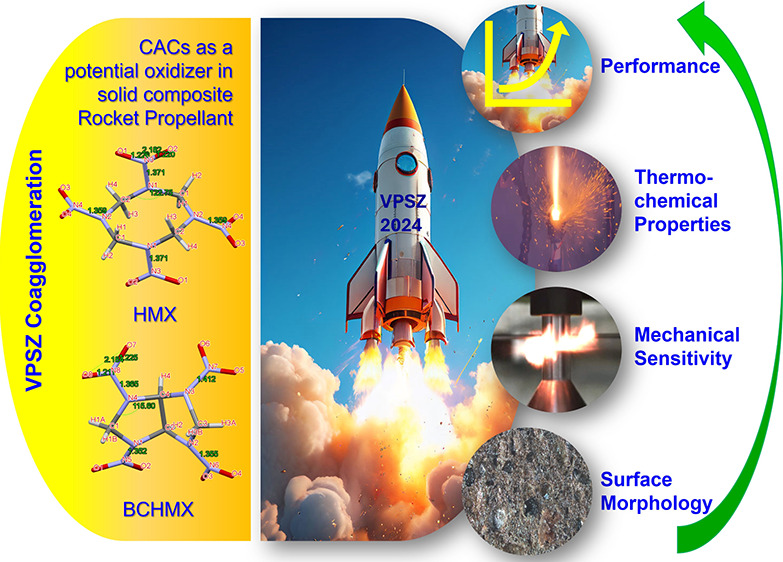

## 1 Introduction

The currently most widely used type of solid rocket propellant comprises a dispersion of finely divided inorganic oxidizer particles (most often ammonium perchlorate—AP) with a powdered metallic propellant (usually aluminum) in an elastomeric binder. Not only in terms of toxicity, especially in demilitarizing propellant grains, but also in reducing or eliminating detection of a flying missile object, procedures are developed to replace AP^1^ (i.e., exclusion of hydrogen chloride from the combustion products^2^). Similarly, for extruded impregnated two-component gun-powders, the replacement of nitroglycerin is a topic currently under investigation. 1,3,5-Trinitro-1,3,5-triazinane (RDX) and especially 1,3,5,7-tetranitro-1,3,5.7-tetrazocane (HMX) are applied as substitutes in the above sense; these nitramines increase the gravimetric specific impulse and/or density specific impulse of the composite propellants, and by this, they also have an increased muzzle velocity of missiles and prevent catastrophic accidents due to unplanned initiation of weapon propellants based on nitrate esters.^3,4^ In the context of the mentioned substitutions, also propellants with dual oxidizers are of interest and practical application; besides, HMX and AP^1^ dual mixtures are studied^4^ with content of 2,4,6,8,10,12-hexanitro-2,4,6,8,10,12-hexaazawurtzitane (CL20) and/or ammonium dinitroamide (ADN) in particular; in a dual AP/HMX oxidizer, the former has a synergistic effect on the thermal decomposition and combustion of the latter one.^1^

Until 2023, the literature (including the monograph from which the chapters^1,4^ were taken) contained no mention of the application of the relatively new and attractive cis-1,3,4,6-tetranitrooctahydroimidazo-[4,5-*d*]­imidazole (BCHMX) in propellants. In our knowledge, this nitramine was developed mainly for this use, in the eighties of the last century, by the group of Prof. Sysolyatin from Biysk in Siberia in the form of a technologically passable procedure with a good yield;^5,7^ however, the corresponding results were classified^6^ for as long as 30 years and a first short information in face of international audience was presented in 2001 ^6^ (see also quotations in paper^5^). Independently from the Russian colleagues, roughly 20 years later, in principle, the same synthesis was developed at University of Pardubice in Czechia,^8,9^ where BCHMX was tested as an active component of the plastic-bonded explosives (PBX).^10^ Mainly due to the complicated synthesis^7,9^ and hydrolytic instability of the intermediate of BCHMX production, i.e., tetrahydrate of the tetrapotassium salt of octahydroimidazo-[4,5-*d*]­imidazol-1,3,4,6-tetrasulfonic acid (TACOS-K),^11^ the developed process is not suitable for a mass production as it is in RDX or HMX. Thus, in the field of explosives, BCHMX could be used only for very special applications (the different charges for police usage, rescue systems mainly in fighter aircraft, etc.), at most as for enhancing the explosive characteristics of PBXs.^12^ As can be seen from the published papers,^5,6^ this nitramine might have been produced in Russia from the beginning of the 1980s. As indicated by the mentioned literature,^6,10,13,14^ this BCHMX nitramine is very important as a modifier for application in propellants (see, for example, the extruded RDX gunpowder ECL120). An information about ECL120 was confirmed by our preliminary attempt in replacing a part of nitroglycerine (NG) in extruded gunpowder by RDX, with a little admixture of BCHMX, when an enhanced effect of this admixture on the powder initiation and burning was registered.^15^ The problem in substituting NG, but also AP, is the usual decrease in the burn rate and specific impulse, solved in papers^14,15^ by increasing the HMX or BCHMX content in the aluminized HTPB propellant or by adding a certain amount of AP to the HMX propellant.^1^

It appears that the structure-molecular specificity of BCHMX^8,10^ in comparison with HMX^10^ and its associated thermochemical properties^10^ could be particularly suitable for the design of heterogeneous propellants with good combustion properties even under subatmospheric pressures. The authors of papers^6,16^ believe that BCHMX will not be as sensitive to combustion catalysts as is the case with HMX. The question remains, however, whether and what effect the addition of BCHMX as a modifier could have on the combustion of an HMX-aluminized HTPB propellant, and this in the form of a mechanical mixture of the two nitramines, compared to the effect of their coagglomerate of BCHMX/HMX (for coagglomerates, see, e.g., ref 17) and all in comparison to pure HMX. This effect has not yet been described in the open literature. Therefore, in this work, we want to verify how the partial replacement of AP in a standard propellant mixture (with HMX as active component) first with pure HMX and then with a mechanical mixture of HMX/BCHMX and then with a coagglomerate of HMX/BCHMX (both with a nitramines mass ratio of 8:1) affects the sensitivity and combustion properties of the propellant. The above activity is carried out in this paper on batches of propellants in which 10, 20, and 30 wt % of AP are replaced by the nitramines.

## 2 Materials

### 2.1 Preparation of Coagglomerated Crystals of HMX/BCHMX (CACs) by the VPSZ Coagglomeration^17^ and Physical Mixture of HMX/BCHMX (PM)

α-HMX and BCHMX were synthesized in the Łukasiewicz Research Network - Institute of Industrial Organic Chemistry in Warsaw. The preparation of CACs (800 g) was carried out according to our previously described VPSZ coagglomeration method:^17a^ 8 wt. parts α-HMX were mixed with 1 wt. part BCHMX and dissolved in dimethyl sulfoxide until a clear solution was formed. From this solution, the nitramines were then precipitated using distilled water, and the coprecipitate was filtered and dried. In the second step, VPSZ coagglomeration, the coprecipitate was stirred in 1000 mL of gently boiling chloroform for 3 h. Preparation of the physical mixture of α-HMX and BCHMX (8:1) has been carried out by mixing both manually in a dry condition.

### 2.2 Preparation of Propellant Batches

Propellant samples were prepared as per Table 1, with each batch size being 700 g. The mixing conditions were 10 min in normal atmospheric and 15 min in vacuum conditions. For coarse AP, it was of 10 and 30 min followed by 5 and 10 min HMX and plasticizer ADO mixing. Three series were prepared with the nitramines content (wt %) as shown in Table 1: series I (10), series II (20), and series III (30), where P1, P2, and P3 are for samples of pure β-HMX, P4, P5, and P6 mean samples containing PM and P7, P8, and P9 samples containing CACs.

**Table 1:** PropellantComposition with Individual Components [Sample Codes]^a^

components/%	series I (10%) [P1, P4, and P7]	series II (20%) [P2, P5, and P8]	series III (30%) [P3, P6, and P9]
AP	56	46	36
HTPB	11	11	11
Al	18	18	18
α-HMX/PM/CACs	10	20	30
FeNPs	1.0	1.0	1.0
curing agent (DDI)	2.1	2.1	2.1
plasticizer (ADO)	1.9	1.9	1.9
total	100%		

a Note: CACs—coagglomerates of HMX/BCHMX, PM—a physical mixture of HMX/BCHMX, DDI—dimeryl diisocyanate, and ADO—dioctyl adipate.

## 3 Results

### 3.1 Spectroscopic Methods

#### 3.1.1 Powder X-ray Diffraction

The undergone polymorphic changes in the CACs of HMX/BCHMX and their physical mixture were analyzed by employing the PXRD technique. The obtained intense 2θ values peaks are shown in Table 2 in comparison with the pure nitramines define and diffractograms in Figure 1. Both coformers show the polymorphic stability after undergoing coagglomeration, with HMX transformed from α to β polymorphic state after interaction with the BCHMX, whereas in the physical mixture, it remains without a change. This indicates that there are interactions between both coformers in the form of weak hydrogen and van der Waals kinds of interactions.

**Figure 1:**
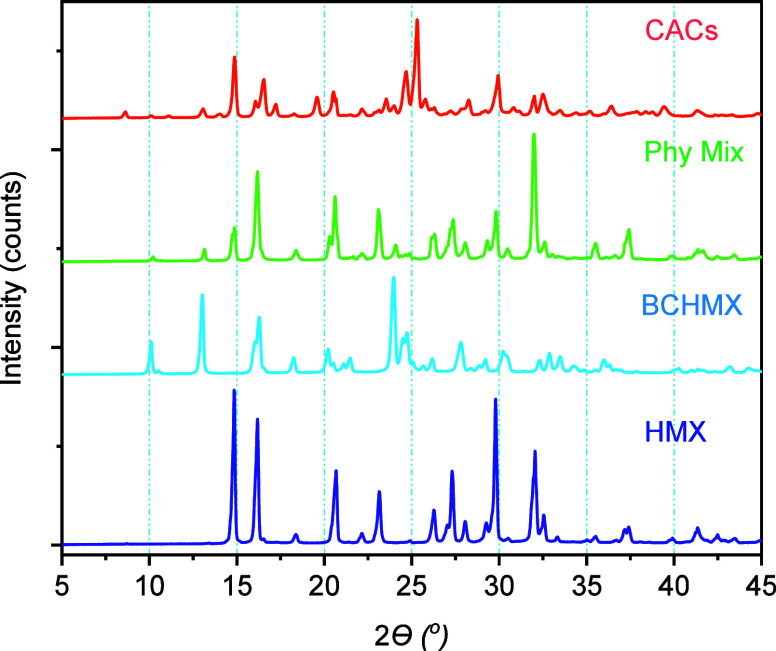
PXRD diffractogram of pure HMX, BCHMX, physical mixture, and CACs.

**Table 2:** PXRD Data for Pure Nitramines and CACs

Sr no.	code design	2θ values for intense peaks/°
1	BCHMX	9.74, 12.65, 23.57
2	α-HMX	14.72, 16.4, 24.54, 29.78
3	β-HMX	14.61, 16.31, 24.45, 25.07, 32.31
4	δ-HMX	13.10, 17.02, 24.34
5	HMX/BCHMX PM	14.86, 16.18, 20.6, 23.08, 27.36, 29.80, 31.98
6	HMX/BCHMX CACs	14.86, 16.54, 20.52, 24.66, 25.30, 29.92, 32.48

#### 3.1.2 Fourier Transform Infrared Spectroscopy (FTIR) and Raman Spectroscopy

These methods were used to analyze the starting nitramines CL20 and HMX, their physical mixture (PM), and coagglomerate (CACs). The results are summarized in the Supporting Information, S1.6. From the obtained FTIR spectra, the changes in intermolecular interactions in CACs compared with PMs and pure nitramines are sufficiently identified (see Table S2). The measured Raman spectra of the CACs and PM, HMX, and BCHMX are summarized in Figure 2 and Table S3 in the Supporting Information. Similarly, as in the case of FTIR, the shifts in CACs crystals, seen in Figure 2, are relatively different from those of PM. This suggests that both coformers are actively involved in intermolecular interactions.

**Figure 2:**
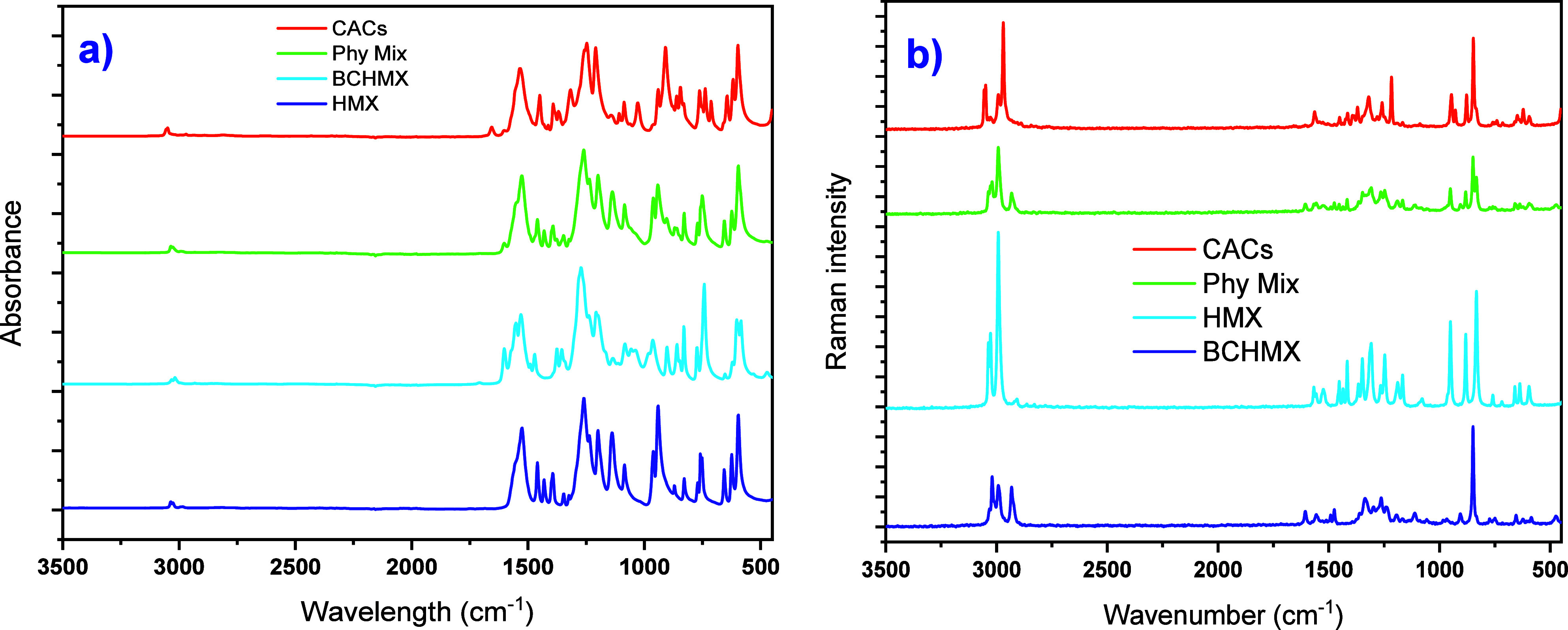
(a) FTIR spectra of physical mixture, CACs, HMX, and BCHMX; (b) Raman spectra of physical mixture, CACs, HMX, and BCHMX.

### 3.2 Morphology and Particle Size Measurements

The morphological changes after VPSZ coagglomeration and propellant samples were analyzed to understand their surface morphology, employing FESEM analysis. Particle size analysis shows (Table 3) that coagglomeration increased the specific surface area somewhat and reduced the size of particles with uniform crystals. To illustrate the dispersion of the applied NAs, propellant samples were prepared for analysis by FESEM and a microscope by cutting them into 3 mm cubes, and the surface was analyzed at one corner and at the edge and surface of these cubes to understand the heterogeneous arrangement of the NAs. The CACs and PM images taken by FESEM are shown in Figure 3a–d. Figure 3a,b shows microcrystals of CACs with rounded edges, while PM in Figure 3c,d represents a mixture of BCHMX microcrystals with relatively sharp edges and the α-HMX porous clusters.

**Table 3:** Particle Size Measurements of Propellant Ingredients

		particle size analysis			
Sr no.	code design	surface area (m^2^ kg^–1^)	Dv (10) μM	Dv (50) μM	Dv (90) μM
1	HMX/BCHMX PM	780.80	3.19	27.60	90.90
2	HMX/BCHMX CACs	904.30	2.70	21.30	97.90
3	HMX	725.00	4.12	28.31	91.57
4	BCHMX	895.30	7.89	7.63	28.51
5	Al (APS7)	240.00	4.10	8.20	15.30
6	coarse AP	85.00	280.0	270.00	120.00
7	fine AP	470.00	90.00	60.00	40.00

**Figure 3:**
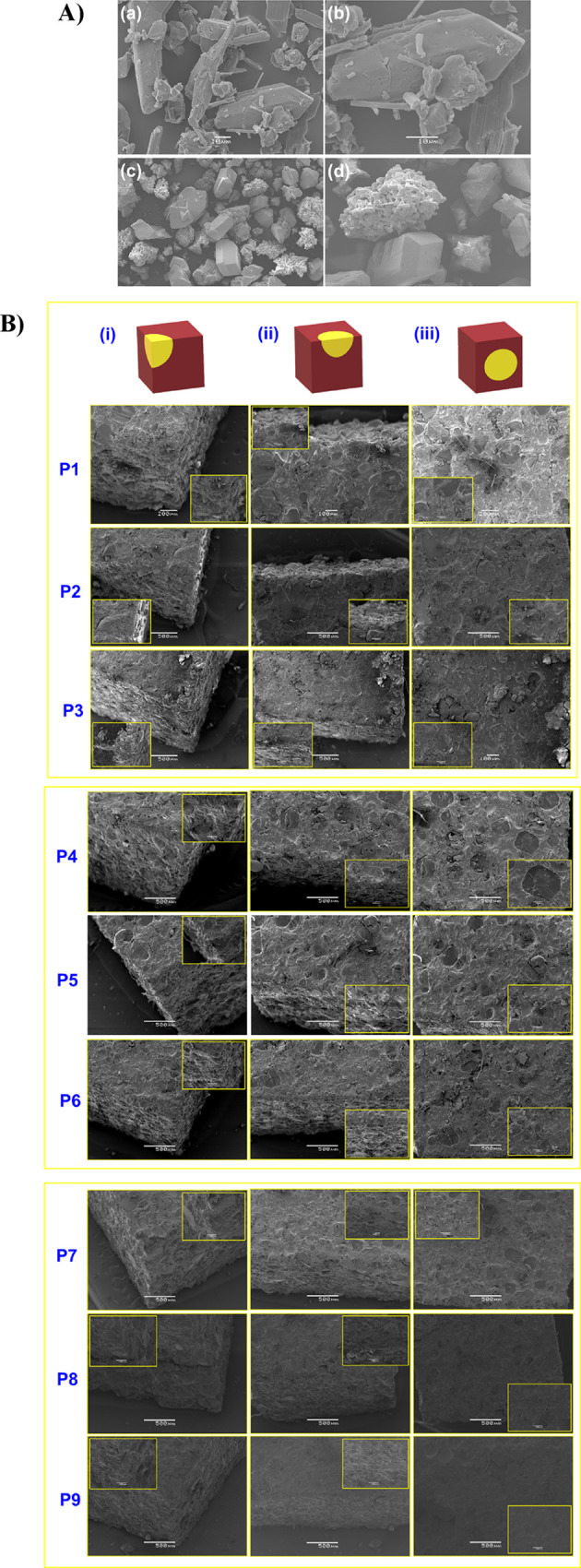
(A) FESEM micrographs: (a, b) CACs and (c, d) PM and (B) images of coarse propellant cubes (P1–P9) 200x with insert 300x at three different sides (i–iii) of propellant cubes.

Both microscopic images (Figure 3) and FESEM images showed that all of the propellant samples exhibited a good distribution of components in the polymeric part of the HTPB. In the former, the distribution of the shining nitramine crystals is well visible. The images in Figure 4 show slight damage to the surface of the crystals (slicing of the samples was done with extra care using a sharp cutting table knife). The distribution of pure HMX crystals on the propellant surface looks good. When the amount of HMX increases (10–30%), its surface morphology becomes slightly harder (Section 3.7 hardness test), which may be due to the arrangement of crystals in the polymer binder with smaller space between them. Similarly, for the physical mixtures (P4–P6), impressions of two types of crystals are clearly visible on the surface of the propellant. In the case of CACs, these distributions are more ordered compared to pure nitramines and PM, and images are taken more quickly (the sample is rapidly charged by the electron beam). This distribution is due to the uniform particle size of CACs, which has also increased the crystal area due to coagglomeration. The mentioned uniform distribution of CACs created more of a “perfect mesh”-type structure that resists needle entry during the hardness penetration test, and the propellant behaves in a “bending rubbery” manner, making samples P7 and P9 impact and friction resistant.

**Figure 4:**
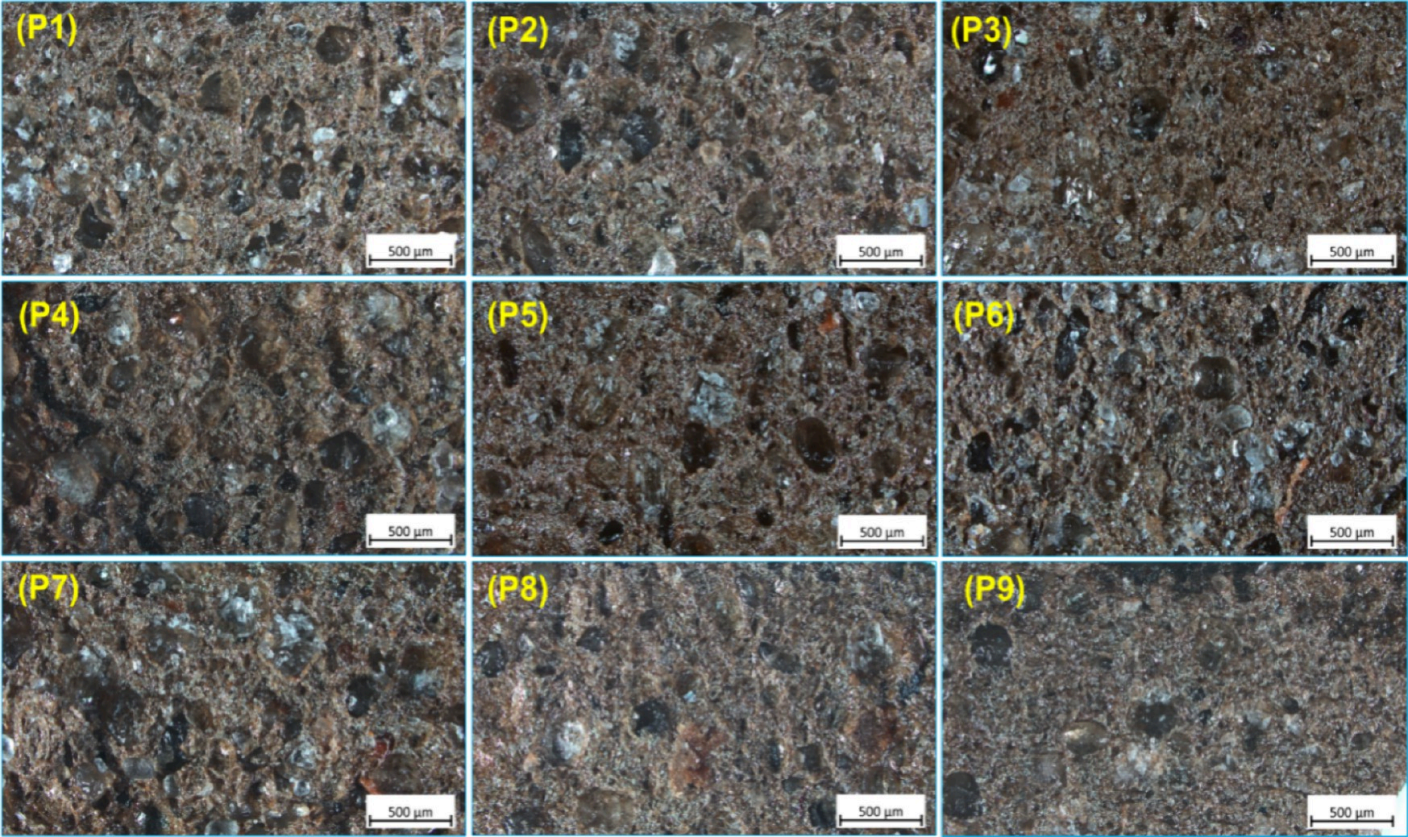
Microscopic images of coarse propellant cubes surfaces (P1–P9).

### 3.3 Thermal Analysis

The thermal stability and influence of the composition of the three types of prepared propellants, including the starting NAs, were specified using a differential thermal analyzer DTA 550 Ex (OZM Research, Czech Republic). The obtained thermograms are shown in Figure 5, and the characteristic changes are summarized in Table 4.

**Table 4:** Summarized Data from the DTA Thermograms of Coformers and Cocrystals with Their Visible Melting Points^a^

		peaks of changes in DTA record/°C (phase modifications)	
sample	melting point/°C	endothermic	exothermic
BCHMX	286 decompn^8^	144	224
HMX	NA	190 (α–δ)	272
ammonium perchlorate (AP)	NA	250	325*, 465
HMX/BCHMX PM	NA	172	232
HMX/BCHMX CACs	NA	NA	230
P1	NA	NA	240*, 271*, 343
P2	NA	NA	237
P3	NA	NA	235
P4	NA	NA	234
P5	NA	NA	225
P6	NA	NA	220
P7	NA	NA	228
P8	NA	NA	225
P9	NA	NA	217

a Note: *minor exothermic peaks.

**Figure 5:**
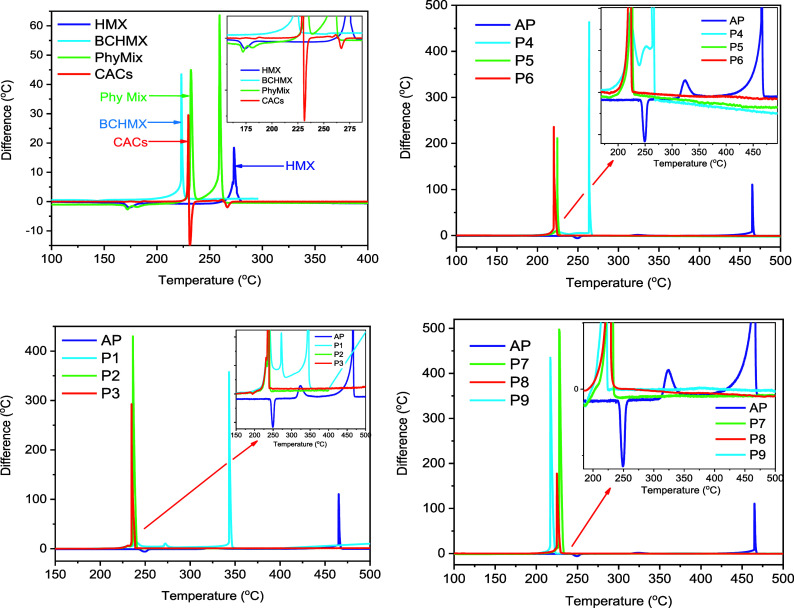
Differential thermal thermograms of pure HMX, pure BCHMX, their physical mixture, their CACs, and propellant batches (P1–P9) in comparison with ammonium perchlorate.

The prepared propellant species exhibited decomposition temperatures between those of BCHMX and HMX except for the P1 and P4 samples. The exopeak temperatures of the propellants decreased with increasing content of NAs in them.

**Table 5:** Results of the 6 h Vacuum Stability Test at 120 °C

	equation of linearization		
sample	slope *k* (kPa g^–1^ min^–1^)	intercept (kPa)	*R* ^2^
PM	0.0010	0.3526	0.9605
	0.0011	0.3373	0.9585
CACs	0.0006	0.2872	0.7556
	0.0005	0.1391	0.9447
P1	0.0006	0.1556	0.9509
	0.0005	0.1298	0.8918
P2	0.0002	0.4990	0.8850
	0.0003	0.3309	0.8944
P3	0.0006	0.3849	0.9395
	0.0005	0.1971	0.9675
P4	0.0011	0.5763	0.9868
	0.0008	0.1748	0.9717
P5	0.0014	0.5011	0.9880
	0.0010	0.3537	0.9694
P6	0.0009	0.3473	0.9748
	0.0014	0.2944	0.9769
P7	0.0005	0.0149	0.9864
	0.0002	0.6334	0.9672
P8	0.0011	0.9688	0.9885
	0.0008	1.2734	0.9685
P9	0.0015	0.1646	0.9732
	0.0010	0.3110	0.9820

### 3.4 Ignition Temperature

The ignition temperature was determined by heating of a 100 mg sample of the given substance at a heating rate of 5 °C min^–1^ until the point of ignition of the sample was reached.^22,23^ This temperature was determined in Wood’s alloy, in Polish Inst. Ind. Org. Chem. using the DTA 551-Rez instrument (OZM Research, Czechia) to control the linear temperature rise of Wood’s alloy; see the Supporting Information, Section S1.12 for details of the instrument and methodology. The observed results are given in Table 6. The decomposition temperature varied with the percentage of nitramines; for propellant samples with pure HMX, compared to those with PM and CACs, the last-named ones had a slightly higher decomposition temperature (±0.5–1 C). However, samples containing both PM and CACs showed a relatively larger dispersion of these temperature values (±10 C) due to the higher reactivity of BCHMX.

**Table 6:** Properties of Propellant Samples^a^

sample codes	hardness (°ShA)	impact sensitivity (J)	friction sensitivity (N)	DMA *T*_g_ (°C)	ignition temperature (°C) (Ψ)
HMX/BCHMX PM	N/A	3.0	80	N/A	230.7
HMX/BCHMX CACs	N/A	5.0	120	N/A	227.6
P1	32.45	17.15	160	–78.5	239.9
P2	32.95	17.15	160	–77.75	239.4
P3	36.56	14.70	120	–78.35	234.6*
P4	34.65	12.25	120	–78.65	223.6
P5	35.65	9.80	120	–78.30	218.9
P6	37.30	9.80	80	–78.65	221.6*
P7	43.05	17.15	160	–78.25	224.9
P8	46.25	14.70	160	–78.65	219.8
P9	47.17	12.25	120	–78.20	221.4*

a Note: *burnt with intense flame, (Ψ) ignition temperatures of all samples measured by Peters apparatus at room temperature (20.1 °C) by visual observation of fire formation.

### 3.5 Vacuum Stability Test STABIL

An upgraded STABIL 16-E STABIL VI instrument was used (manufacturer OZM Research, Czechia—the original instrument is described in paper^18^). The output of the isothermal measurements in our case is the time dependence of the pressure evolution of the decomposition products at 120 °C.

By linearizing this dependence for an isothermal exposure of 60–360 min, the data were obtained, which are summarized in Table 5; the slopes of these lines, *k*, correspond to the reaction rate of evolution of the gaseous products from a zero-order reaction^19,20^ —these values of *k* therefore represent the specific rate constant (i.e., zero-order reaction constant—the values of *k* are in kPa g^–1^min^–1^).

### 3.6 Dynamic Mechanical Analysis (DMA-Tg)

The glass transition temperature, *T*_g_ *,* is a phenomenon of amorphous polymers. At this temperature, polymers undergo a transition from a glassy state to a rubbery state. This information is often used for quality control, predicting product properties, and informing processing conditions or thermal history. For our purposes in this work, we used a TA dynamic mechanical analyzer, the DMA850 (TA Instruments, New Castle, DE, USA), see in the Supporting Information, S1.13 for details of the instrumentation and the results obtained are shown in Table 6.

### 3.7 Hardness Test

After curing the propellant samples, its hardness was assessed using a Shore A hardness tester according to ASTM D 2240 ^21^ (average of six measurements at different locations on the surface); the results obtained are given in Table 6. For all samples, the insertion of nitramines increased the hardness of the propellant samples. In the case of pure HMX, the driving gas was softer compared to that of the insertion of the physical mixture HMX/BCHMX or CACs.

### 3.8 Impact and Friction Sensitivities

The sensitivity was determined employing a standard impact tester with exchangeable anvil (Julius Peters^22–24^ ). Friction sensitivity was tested in the Peters apparatus according to the method European standard PN-EN 13631-4 2003 (details of both these methodologies and instrumental techniques, see the Supporting Information, S1.10 and S1.11). The observed results are listed in Table 6.

### 3.9 Burning Rate Measurements

The dependence of the burning rate on pressure was measured by using an SV2 (Stojan Vessel, OZM Research, Czechia) closed vessel. ABSW software (OZM Research) was used to evaluate the measurement results; the evaluation is based on pressure traces as shown in eq 1.^25^ The uncertainty of the pressure measurement is within 0.5% as given by the manufacturer.

$$u(p)=\left(\frac{e_{0}}{\left(P_{\max}-P_{z}\right)}\right)\times \left(\frac{dp}{dt}\right)$$ 1

where *e*_0_ is the unit burning thickness of the solid propellant grain, *P*_max_ is the maximal pressure in a closed bomb, *P*_*z*_ is the ignition pressure, and $\left(\frac{dp}{dt}\right)$ is the pressure derivative as a function of time. Details of the measurement procedure are described in the Supporting Information (Section S1.15).

The specification of the propellant burning characteristic for a particular composition depends on only the chamber pressure and initial temperature of the propellant. For rocket propellants, the form known as Saint Robert’s or Veille’s law is chosen since combustion pressures rarely reach 2000 psia (13.8 MPa):^26^

$$r=aP^{n}(P<2000\;\mathrm{psia})$$ 2

where *a* is a constant dependent upon the propellant nature and initial temperature, *P* is pressure, and *n* is the pressure exponent. High-pressure exponent in this work means that *n* is larger than 0.6.^27^

The linear burning rate is the rate, *r*, at which the burning surface recedes along the normal to the surface.^28^ In the present investigation, the pressure range 3.0–20.0 MPa is considered (see Figure 6 and Table 7). The burning rate was calculated using just the linear portion of the burn process. At this pressure, transient burning was observed, which would account for the faster burning rate.

**Table 7:** Burning Rate of CACs Propellants Are Reported Compared to Earlier Literature Works^a^

Sr no.	compound	amount (%)	pressure range (MPa)	constant (*a*)	pressure exponent (*n*)	burning rate (mm/s)	ref
1	AN/crown ether AN–benzo-18-crown-6 (B18C6)	0.2	0.50–7.00	0.130	0.62	0.59	^28^
2	HMX/CL20 CC	NA	0.69–13.80	0.519	0.782	2.35	^29^
3	HMX/CL20 PM	NA	0.69–13.80	0.474	0.771	2.1	^29^
4	TNT/CL20 CC	NA	0.69–13.80	0.284	0.767	1.25	^29^
5	TNT/CL20 PM	NA	0.69–13.00	0.228	0.955	1.44	^29^
6	CL20/HP solvate	NA	1.38–6.89	0.507	0.774	2.26	^29^
7	HMX/AP composite	NA	0.69–13.80	0.463	0.668	1.68	^29^
8	HMX/AP coarse PM	NA	0.69–13.80	0.360	0.697	1.38	^29^
9	HMX/AP fine PM	NA	0.69–13.80	0.530	0.642	1.83	^29^
10	TNT	NA	3.45–13.80	0.085	0.803	0.4	^29^
11	HMX, Atwood et al.	NA	0.69–10.30	0.236	0.816	1.14	^29^
12	HMX, Sinditskii et al	NA	0.2–10.00	0.25	0.81	1.19	^29^
13	CL20, Atwood et al.	NA	0.69–10.30	0.526	0.744	2.21	^29^
14	CL20, Yang et al.	NA	3.00–9.00	0.491	0.846	25.1	^29^
15	TNT, Kondrikov et al.	NA	5.00–58.00	0.217	0.422	0.49	^29^
28	AP/AN CCs	AN (28–68) AN (28) AP (constant)	1.00–7.00	NA	(0.55–0.70 ± 0.05)	5.00–6.00*	^35^
29	HMX	55–80%	0.40–10.00	NA	0.50–0.90	0.50–7.00*	^30^
30	FOX7/RDX-BuNENA - 1	FOX7 (5–50) RDX (0)	0.5–14	NA	NIL	NIL	^36^
31	FOX7/RDX-BuNENA - 2	FOX7 (5–30) RDX (20)	0.5–14	NA	0.86	5.44	^36^
32	FOX7/RDX-BuNENA - 3	FOX7 (20) RDX (10–30)	0.5–14	NA	93	5.4	^36^
33	DB matrix/RDX	(88/22)	0.5–14	NA	NA	8.6–10.6*	^36^
34	DB matrix/RDX/Al	(85/12/3)	0.5–14	NA	NA	9.2–10.7*	^36^
35	DB matrix/RDX/AP/ZrSiO_3_	(80/12/6/2)	0.5–14	NA	NA	9.8–11.5*	^36^
36	DB matrix/RDX/AP/Al	(79/12/6/3)	0.5–14	NA	NA	10.4–12.5*	^36^
37	DB matrix/RDX/AP	(79/12/9)	0.5–14	NA	NA	10.4–11.7*	^36^
38	HMX	10–30	4.00–14.00	NA	0.6–0.9	4.50–8.00*	^14^
39	BCHMX	10–30	4.00–14.00	NA	0.65–0.8	4.50–8.00*	^14^
40	P1	10	3.00–20.00	3.09	0.47	7.73	CW
41	P2	20	3.00–20.00	2.36	0.53	6.73	CW
42	P3	30	3.00–20.00	1.89	0.62	6.13	CW
43	P4	10	3.00–20.00	2.98	0.49	7.81	CW
44	P5	20	3.00–20.00	2.47	0.55	7.35	CW
45	P6	30	3.00–20.00	2.01	0.60	6.12	CW
46	P7	10	3.00–20.00	2.96	0.60**	8.44	CW
47	P8	20	3.00–20.00	2.63	0.56	8.01	CW
48	P9	30	3.00–20.00	2.21	0.54	7.01	CW

a Note: the 95% confidence bounds for each fit parameter are also given. The fit parameters from the literature were either directly reported or calculated.^30–34^ CW—results of current work. Sr nos. 38–48 were considered burn rates at standard pressure 6.89 MPa. *the range of values, because of they made a lot of compositions with minor changes in compositions. **measured in the pressure range 3–17 MPa due to limited amount of sample.

**Figure 6:**
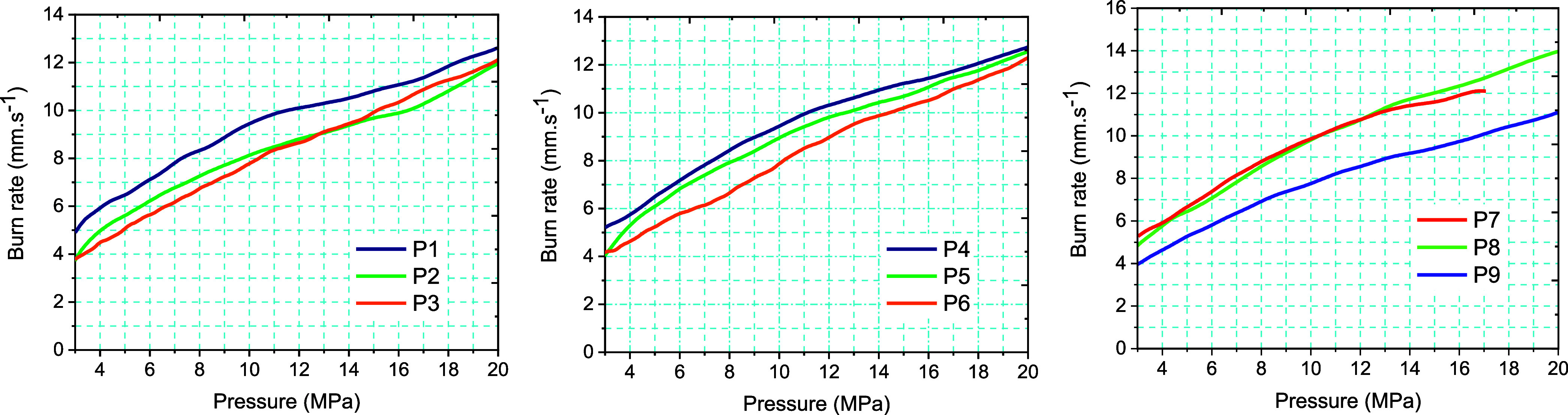
Measured steady burning rates of the HMX, HMX/BC PM, and HMX/BCHMX CACs (sample P7 measured in the pressure range 3–17 MPa due to a limited amount of sample).

Furthermore, Table 7 displays the Saint Robert’s law burning rate fit parameters with 95% confidence ranges for each of the investigation’s materials reported in the literature with current CACs propellant for comparisons.^29^

Moderately larger and uniform particles are typically preferred over nano or submicron explosive particles due to their low critical diameter, high burning rate, and detonation rate.^37^ When it comes to increasing the rate at which solid propellants burn, ultrafine explosive particles have an advantage over bigger ones,^32,33^ which is well achieved in case of CACs.

### 3.10 REAL Software Calculations

Prediction of specific impulse, flame temperature, and calculations of oxygen balance was conducted resorting to the software REAL,^38^ which is a thermodynamic code that is used for computer simulation of chemical equilibrium in complex chemically reacting systems. Enthalpies of formation and the propellant sample components were used as input data (see the Supporting Information, Section S1.2, Table S1 with the characteristics of the propellant components required for calculations). Standard pressures of 7 MPa in the chamber to 0.1 MPa at expansion out of the nozzle were used for calculations. For all specific impulse calculations, the virtual equation of state was used (Table 8).

**Table 8:** Results of Thermodynamic Performance Calculations of the Studied Propellants

properties/sample no.	P1	P2	P3	P4	P5	P6	P7	P8	P9
oxygen balance (%)	–45.91	–51.48	–57.04	–45.86	–51.37	–56.89	–45.86	–51.37	–56.89
density experim. (g cm^–3^)	1.7462	1.7454	1.7425	1.7475	1.7430	1.7335	1.7445	1.7362	1.7355
specific impulse (s)	262.72	257.86	253.76	262.8	258.0	253.87	262.8	258.0	253.9
heat of combustion (J g^–1^)	8258.45	8317.83	8377.31	8265.19	8331.32	8397.55	8265.37	8331.66	8398.07
adiabatic flame temperature (K)	3168	2987	2710	3172	2994	2720	3172	2994	2720
adiabatic flame temperature (°C)	2895	2714	2437	2869	2721	2447	2899	2721	2447

## 4 Discussion

### 4.1 Coagglomeration

The VPSZ coagglomeration of HMX with BCHMX resulted in microcrystals—CACs with a larger specific surface area as was for their physical mixture (see Table 3 and Figure 3a–d)—an increase in specific surface area by undergoing coagglomeration is common.^17^ Compared to the physical mixture, the obtained CACs have rounded edges. In the FESEM images (Figure 3a–d), porous crystals of crude HMX are clearly observed, which disappeared during coagglomeration.

### 4.2 Initiation Reactivity Characteristics

#### 4.2.1 Differential Thermal Analysis (DTA)

The intercomparison of CACs and their physical mixtures HMX/BCHMX in Figure 5 showed that CACs undergo a one-step clear decomposition, while this PM exhibits two exopeaks (the first one is induced by BCHMX decomposition). Both the physical mixture PM and CACs have exothermic peaks somewhat shifted to higher temperatures compared to those of pure BCHMX. This behavior of CACs in particular is consistent with the results of spectral monitoring of these mixed crystals, i.e., with the nature of the intermolecular interactions between the two conformers (see also paper^17^). This is particularly evident for the third type of propellant sample with 30 wt % nitramine abundance (P7–P9), where CACs contributed to more efficient decomposition changes around 218 ± 10 °C for all three concentrations in contrast to the other sample types (P1–P8).

For pure AP, an endothermic peak is observed at about 250 °C, which is assigned to the crystallographic transition of AP from orthorhombic to cubic.^39^ By further heating, AP underwent two complicated decomposition stages,^40^ i.e., a low-temperature stage at 329.3 °C and a high-temperature stage at 435.5 °C, which are followed by two exothermic peaks. The mentioned changes of AP during heating are clearly shown in Figure 5.

The synergistic effect of AP on the decomposition and combustion of HMX^1,41^ is clearly evident from the decomposition curve of the driving gases P1 (with 10% HMX) and P4 (with 10% PM), which starts in the temperature region of the polymorphic transition of AP and consists of three exothermic peaks (see Figure 5). At higher nitramine contents in the samples and also for P7 with 10% CAC, this effect no longer appears. This also suggests that AP should not have a significant effect on the decomposition of BCHMX. Both the thermal decomposition of HTPB/AP and HTPB/HMX split into two phases with some amount of carbon residue.^42^ Compared to the HTPB/AP mixture, more HTPB and HMX participate in this decomposition and hydrocarbons containing primary amines are formed.^42^ Considering these changes, CACs in the third species (P7–P9) showed more pronounced changes in decomposition for all three concentrations, which is logical considering content of the 30 wt % nitramine. It is worth noting the lower exopicks values for the samples containing CACs, the more thermally reactive BCHMX, introduced into the HMX crystal lattice, also additionally behaves here as an impurity (it is characteristic of nitramine cocrystals^17^).

#### 4.2.2 Ignition Temperature

The initiation reactivity of EMs is related to their energy content, which can be represented by the enthalpy of formation but also by the heat of combustion, *Q*_*c*_ *.* ^19,20,43^ It should be noted that as the AP content of the propellant decreases, the heat of combustion increases (Scheme 1). Thus, the explosion temperature decreases. One from the characteristics of the initiation reactivity is ignition temperature, which should be related to the heat of combustion (presented by Figure 7a—a similar dependence for the double-base propellant is hinted in ref 44). Here, lines P1–P2–P3 correspond to expectation (increasing content of HMX leads to increased reactivity). Figure 7a also shows that the admixture of BCHMX increased the thermal reactivity of samples containing PM and CACs relatively significantly, especially in the case of samples P5 and P8, between which there is quite a big difference in the *Q*_c_ values. Also, the increasing *Q*_c_ values of the samples in the order P3–P6–P9 (they contain of 30% wt. nitramines) are related to problem of the precisely definition of the thermodynamic state of final products of combustion (mainly of the amount of chlorine acidic derivatives),^40^ the compounds of which might perhaps influence the AP effect on the combustion of HMX and HTBP (see paper^42^) even in an oxygen atmosphere (in any case, the presence of BCHMX changes decomposition of these propellants, as it was already mentioned in Section 4.2.1).

**Scheme 1:**
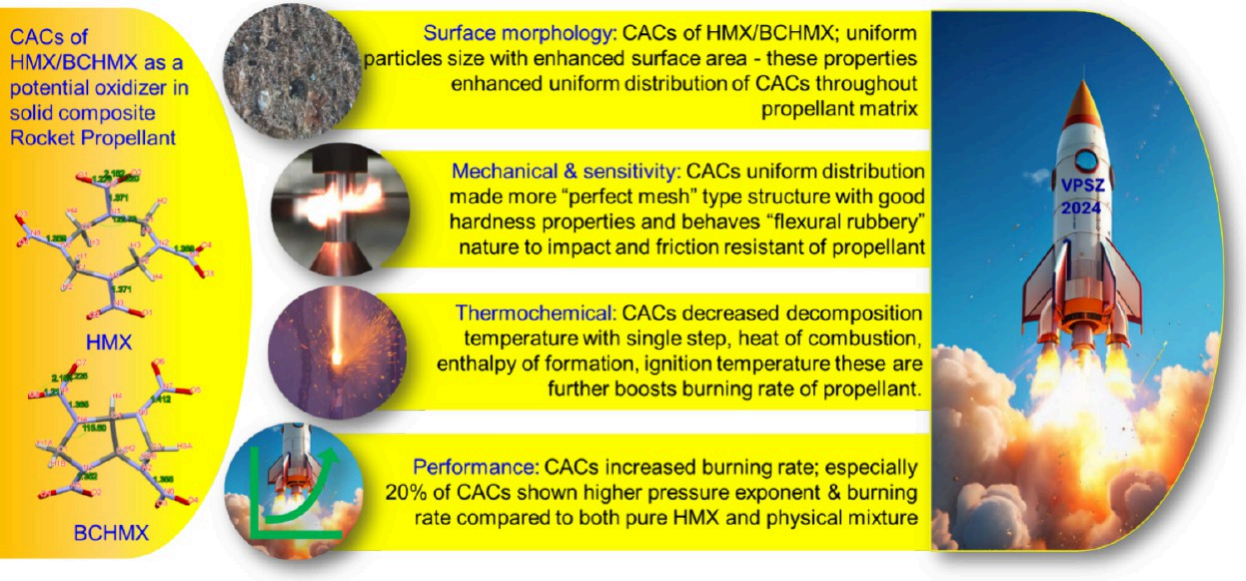
Summarized Results in the Schematic Form (Photograph Courtesy V. B. Patil Specially Prepared for This Manuscript; Copyright 2025); Note: Rocket Picture Was Generated from Free Resource Microsoft Copilot and Remaining Real Time Images from Our Laboratory

**Figure 7:**
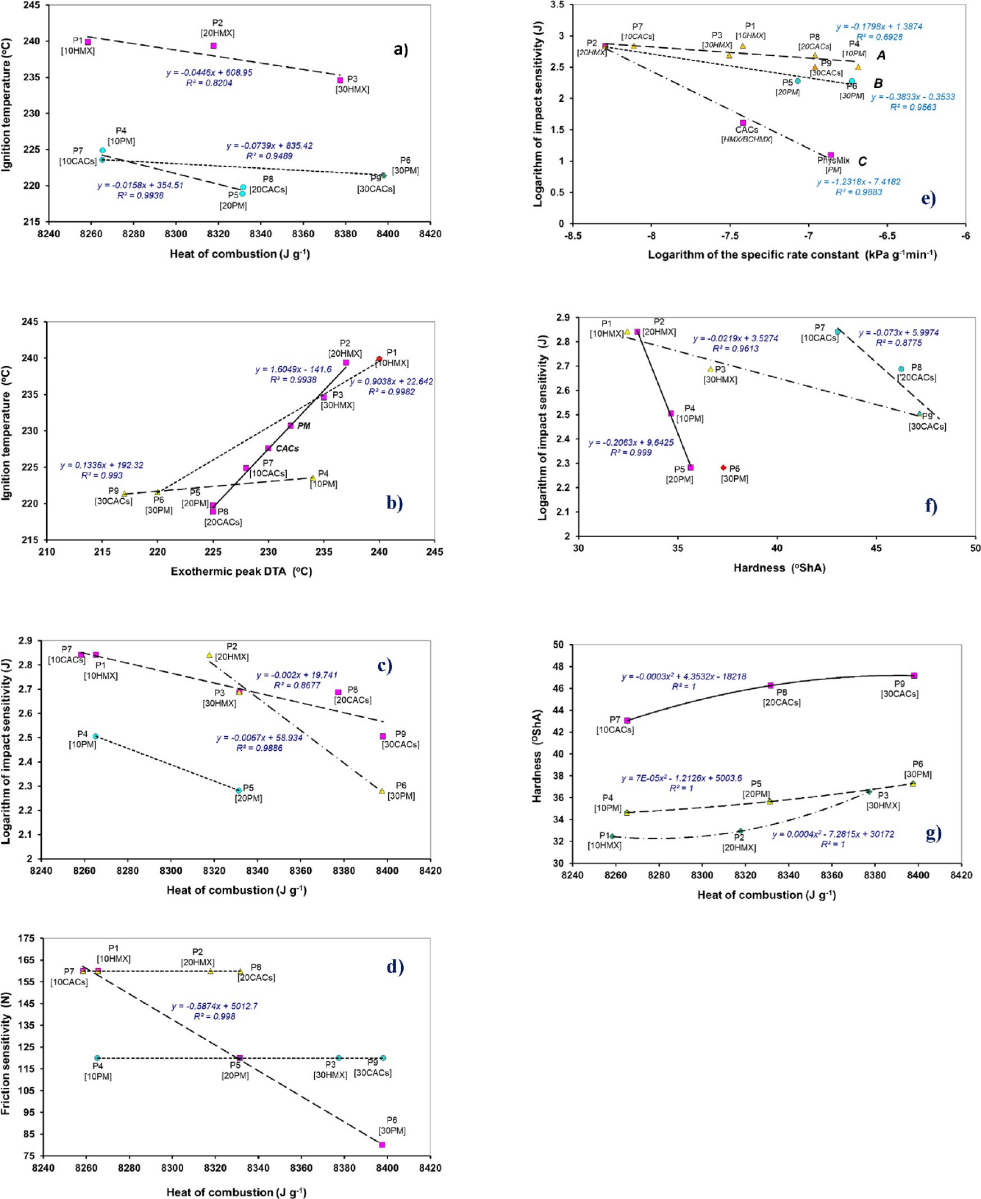
(a–g) Relation of the observed initiation reactivity characteristics of the studied propellants to their physical properties (the number in square brackets below the sample code indicates the percentage of HMX or PM or CACs in the sample): (a) relation of ignition temperatures to temperature of the DTA exothermic peaks, (b) ignition temperature versus heat of combustion, (c) impact sensitivity versus heat of combustion, (d) friction sensitivity versus heat of combustion, (e) mutual logarithmic relationship of impact sensitivity and specific rate constant, (f) semilogarithmic relationship between impact sensitivity and hardness, and (g) relationship between hardness and heat of combustion.

For high explosives, there is a uniform linear dependence between the ignition temperature and temperatures of the exothermic peaks of their decomposition.^45^ For propellants, these two quantities were found to be mutually close in value.^44^ In the case of the propellants studied, this dependence (see Figure 7b) breaks down into three ones; in particular, mainly the dependence for PM-containing samples is deviating. This may be due to the difference in sample weights of the two thermostability tests, but mainly to the as-yet unknown effect of AP on the thermal stability of “free” BCHMX in PM (in CAC, this nitramine is as if protected in the form of a cocrystal—it is a new physical entity also with a new reactivity.).

#### 4.2.3 Vacuum Stability Test

As shown in Table 5, the reproducibility of the selected approach to the VST application is not the best in the PM 30% case, in comparison, for example, with the Russian Manometric Method (see paper^46^). Figure 7e shows the decrease in specific rate constants with decreasing heat of combustion, with the group of samples divided into two subgroups according to nitramine content (see Figure 7e). In particular, the linearization of the decomposition curves for propellants obtained these values. In pure HMX propellants, the lower specific rate constant might be due to well crystal distribution over propellant coarse; similarly, in the case of CACs, after coagglomeration, crystal sizes were unified and made well distributed. However, whereas PM might be due to two kinds of crystals, different sizes of HMX and BCHMX are not well distributed, so the specific rate constant is higher in all PM propellants (P4–P6).^47^ This also shows that CACs, like α-HMX, thermally behave as a single molecule, which means that there will be good interactions between HMX and BCHMX molecules in CACs through intermolecular interactions.^17^

#### 4.2.4 Impact and Friction Sensitivity, and Hardness Test

As can be seen from Table 6, Figure 7c,d, and Scheme 1, the sensitivity to mechanical stimuli of the prepared propellants is mainly influenced by the nature and amount of nitramines incorporated into them (in microcrystalline PM and CACs is reflected physical state of these compositions; see Table 6 and Figure 7c,d); while in the case of pure HMX, its content in the mixture does not contribute significantly to this sensitivity (with perhaps of an exception of 30% wt. content); the opposite is true for PM and CACs. Mainly, PM has a negative effect on both sensitivities, CACs only on the impact sensitivity. Unlike similar mixtures in paper,^14^ where the AP/HTPB/Al propellant matrix significantly increases the sensitivity of the mixed HMX and BCHMX, in studied case, this matrix acts as a phlegmatizer. The logarithmic relationship between impact sensitivity and specific rate constants has already been described^20^ and logically presents an increase in impact sensitivity with increasing values of this constant; in presented case, this relationship is represented by Figure 7e: here, physical mixtures (PM) have a negative effect on the impact sensitivity also incorporated in the propellant (see mainly line B), but increasing the CACs content in the propellant has a similar logically effect—although the latter case is not very significant (line A).

According to the friction sensitivity (FS), the studied samples are quite sharply divided into two groups (see Figure 7d), namely, P1, P2, P7, and P8 and P3, P4, P5, and P9; logically, the samples with PM and also with 30% nitramine have increased FS values.

Hardness studies of propellants coarse indicated that physical mixtures give their hardness in not much varied compared to content of pure HMX, which may be due to both particles BCHMX and HMX distributed randomly in a propellant coarse (as shown in Figure 4). It also indicates its bigger dependence on the HMX content rather than of the BCHMX one. In contrast, the distribution of CAC microcrystals is thus ordered in the material and is bound very tightly. With both the PM and CACs contents, the propellant has a rubbery character. However, in the case of CACs with uniform crystal shape and structure, the propellant is more tightly arranged, more rigid. Increasing rigidity (hardness) of the propellant and its nitramine content corresponds to an increase in its impact sensitivity; this increase being mostly pronounced in samples with PM and the lea of all pronounced in samples with CACs (see Figure 7f). In friction sensitivity tests, the samples with CACs performed better than those with PM (in the last due to the “free” BCHMX crystal content).

Here, Figure 7g presents as a documentation of the dependence of hardness (may be a representant of the nitramine content) of the samples on the heat of combustion (i.e., on a representant of energetic content of EMs in general); it appears that the CACs admixture are the best in this respect.

### 4.3 Dynamic Mechanical Analysis (DMA-Tg)

From the results of the *T*_g_ values determination in Table 7 and Scheme 1, they appear to be essentially the same for all propellant samples. However, according to the *T*_g_ values, the studied samples can be roughly divided into two groups: P3, P5, P7, and P9, and P1, P4, P6, and P8. The P2 sample is the outlier in terms of the *T*_g_ value, but samples P4 (10% PM), P6 (30% PM), and P8 (20% CACs), in this sense, seem to be the best.

### 4.4 Spectral Study

PXRD diffractograms (see Figure 1 and Table 2) show a new crystal phase where HMX changes from α to β polymorphic state upon the interaction with BCHMX, while the original α-HMX remains in the physical mixture. This is confirmed also by the FTIR (Figure 2) and Raman spectra, both in the Supporting Information, S1.6. This implies that there should be interactions between the two coformers in CACs in the form of weak hydrogen and van der Waals interactions, which further caused the above polymorphic changes of HMX. By this are created the conditions for the formation of cocrystals during the coagglomeration process and also affecting the morphological changes of the obtained crystals of uniform size.

### 4.5 Outputs of Burning Rate Measurements

In the literature, data are available on the comparison of the burning rate of cocrystal-filled propellants with the burning rate of their coformers:^30–32^ the burning rate of the HMX/CL20 mixture and cocrystal was similar to that of CL20, regardless of the difference in burning rate between them. The burning rate of cocrystal TNT/CL20 was between the burning rates of its coformers, but cocrystal HMX/CL20 and solvate CL20/HP burned the same as CL20.^29^ The current investigation clearly showed that the application of CACs, compared to pure HMX, and PM leads to a higher burning rate of the propellant. When it comes to increasing the rate at which solid propellants burn in general, ultrafine explosive particles have an advantage over bigger ones,^31,33^ which is well achieved in the case of CACs. All HMX/AP compounds burn faster than HMX or AP alone, and particle size affects burning rate.^29^

In this investigation, the three concentrations of nitramine used differed in the burn rate, with 30% of the contents showing a significant reduction in the burn rate. In contrast, the 10 and 20% wt. CACs content showed a higher burning rate compared to both pure HMX and a physical mixture (PM). According to the previously mentioned effect of particle size on burning, CACs showed a more suitable particle size (see Section 4.2.4) and surface morphology. This aided in the uniform distribution of crystals in the mass and the actual flame burning on the surface of CACs compared to PM and pure HMX. This is because the CACs reached a more uniform particle size and surface area during coagglomeration (Figure 4), which caused them to spread equally over a thick layer of the propellant. Regarding the actual addition of BCHMX and its effect on the propellant performance, it can be seen that BCHMX increased the burning rate of both the physical mixture and CACs compared to that of pure HMX.

The current investigation showed a better burn rate compared to earlier publications (although due to the influence of many factors on combustion, a full comparison is not possible, see Table 7). Anyway, with the same propellant composition, CACs show overall better performance.

As with other energetic materials, an increase in the energy content of the propellant corresponds to an increase in its initiation reactivity, as already shown in Figure 8a (raising in *Q*_c_ corresponds to a decrease in the heat of formation). According to Figure 7e, crystalline CACs are more resistant to thermal decomposition and impact (Table 6) compared to a physical mixture of crystals (PM), but its incorporation into the propellant increases its burning rate more significantly (Figure 8a) than pure HMX or mixture PM. Interestingly, the series II composites (with 20% nitramine) show the opposite pattern of relationship for HMX and PM admixtures in Figure 8a; as already mentioned, this series has the most suitable set of thermochemical parameters for propellant combustion.

**Figure 8:**
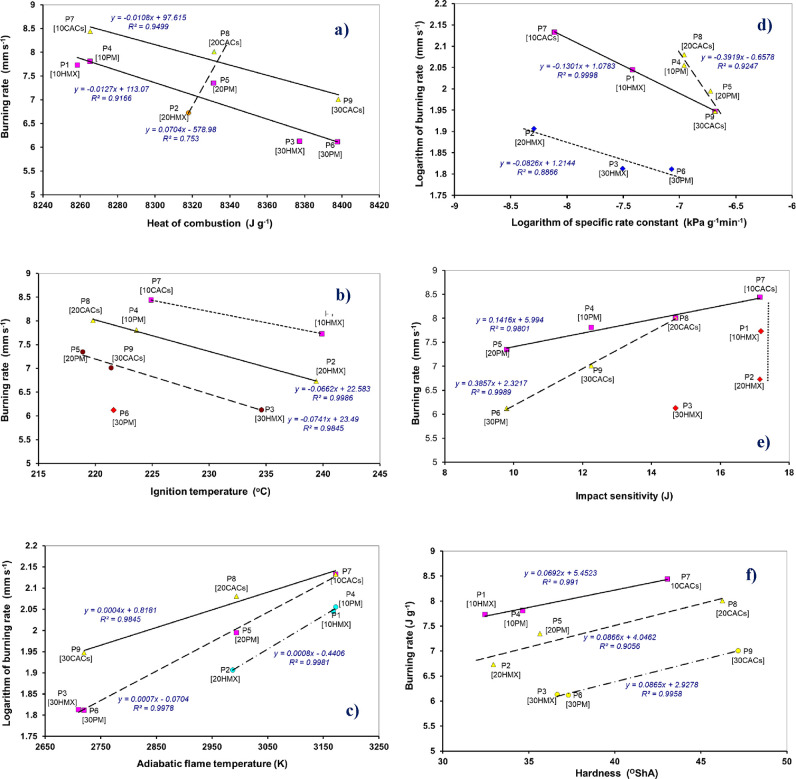
Relationships derived for the burning rate (the number in square brackets below the sample code indicates the percentage of HMX or PM or CACs in the sample): (a) relationship between the burning rate and combustion heat, (b) relationship of the burning rate and ignition temperature, (c) semilogarithmic relationship between the burning rate and adiabatic flame temperature, (d) logarithmic relationship of the burning rate and specific rate constant, (e) relationship between the burning rate and impact sensitivity, and (f) relationship of the burning rate and hardness.

Relationships between the burning rate and propellants reactivities, here ignition temperature, specific rate constant, and impact sensitivity, are presented in Figure 8b–e. An increase in thermal stability, here, the ignition temperature, corresponds, as expected, to a decrease in the burning rate (Figure 8b); a possible relationship between the steady-state burning rate and ignition temperature was investigated several decades ago, but no conclusive evidence has yet been found. However, the trend corresponding to Figure 8b might demonstrate the results of the papers,^48,49^ in which an increase in the burning rate with a decrease in the ignition temperature was observed in the development of the graphene-modified nitromethane monopropellant^48^ or the fuel-rich composite propellant (HTPB/AP).^49^ For the relation with a negative slope, it seems logical that both the sensitivity and burning rate increase with increasing HMX content in the propellant. Similarly, Figure 8c presents a logical semilogarithmic relationship of the burning rate and adiabatic flame temperature, again clearly documenting the significant activating effect of the propellant burning by the CACs admixture compared to the addition of PM or pure HMX.

Concerning relationship of the specific rate constant of isothermal decomposition to burning rate (for the logarithmic version, see Figure 8d), the opposite trend is found comparing with Figure 8b,c (the same is true for the analogous relationship for the detonation rate of EMs^20^): the burning rate increases as this constant decreases, with the three highly filled samples (P2, P3, and P6) being separated from the other propellant samples in terms of Figure 8c. In the case of impact sensitivity (Figure 8e), the increase in burning rate corresponds to a decrease in impact sensitivity (samples with pure HMX do not correlate with this dependence), which corresponds to the relationship in Figure 7c. The effect of hardness on combustion is illustrated in Figure 8f: increasing hardness and decreasing nitramine content in the propellant correspond to increasing burning rates, with an inherent dependence for each nitramine content.

#### 4.5.1 Relations of the Propulsion Characteristics

A very important mutual comparison of impact sensitivity and pressure exponent (Figure 9a) documents that BCHMX in the form of its coaggloerate (cocrystal) with HMX with its increasing amount in the propellant mass reduces the pressure exponent, thus making this composition suitable for rocket propellant applications. Pure HMX and the physical mixture (PM) of BCHMX with HMX do not have this property. The same effect of CACs can be found in Figure 9b, i.e., in the relationship between the pressure exponent and propellant hardness. Here, for the most efficient series II samples, a straight line can be interpolated through the data P2, P5, and P8. It is also clear that the incorporation of this series into the propellant mass increases significantly its hardness

**Figure 9:**
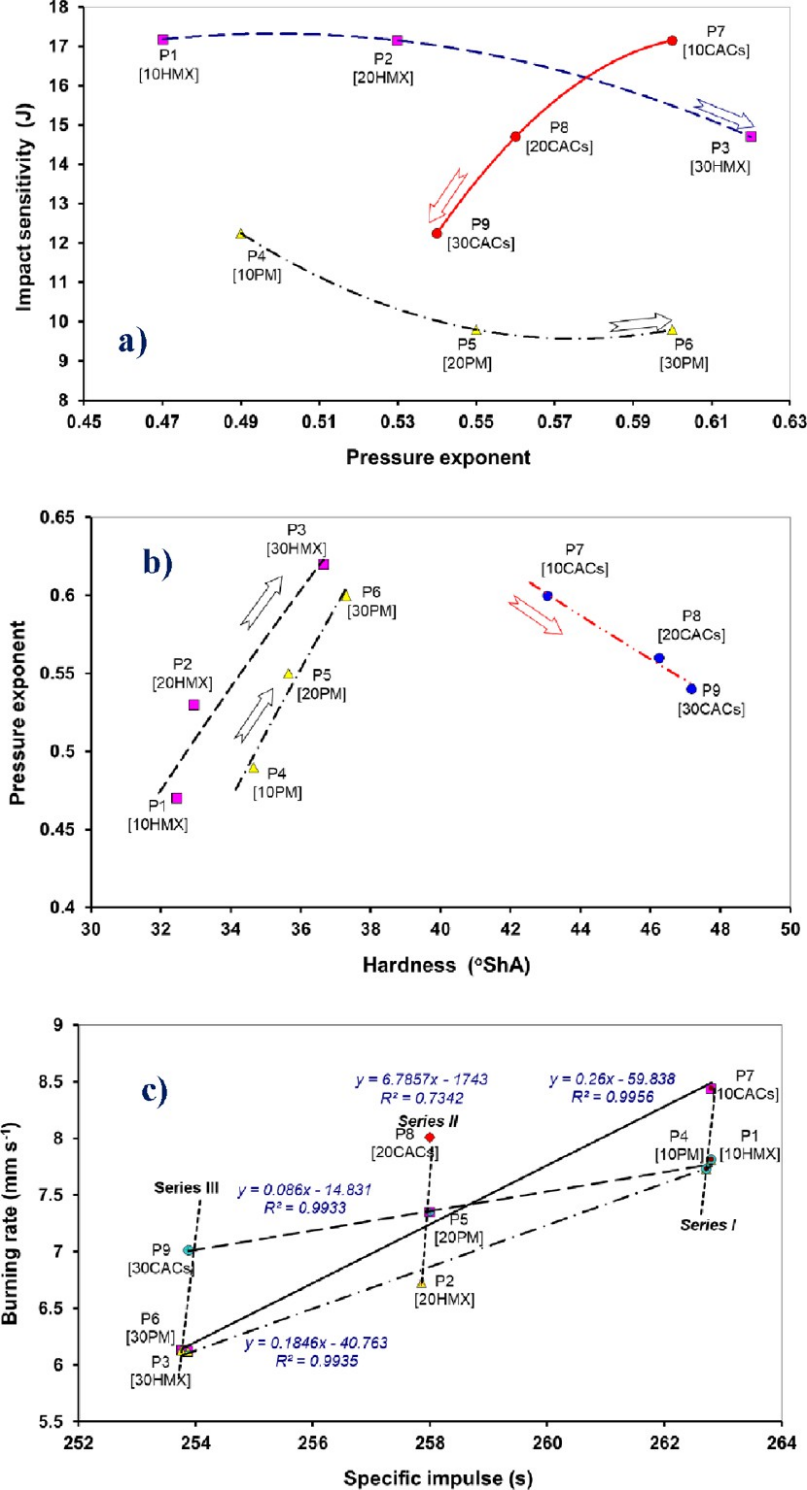
Relationships of propulsion characteristics (the number in square brackets below the sample code indicates the percentage of HMX or PM or CACs in the sample): (a) trend diagram of the impact sensitivity and pressure exponent relationships, (b) trend diagram of the pressure exponent and hardness relationship, and (c) relationship between burning rate and specific impulse.

The correlation of the burn rate and specific impulse in Figure 9c shows both the known decrease in impulse with the degree of AP nitramine replacement and the significantly positive effect of CACs on the burn rate. This comparison looks like a linear dependence, but for hybrid rocket propulsion, it has a polynomial shape;^50^ the authors of the cited paper cite as the novelty of their study the formulation of this polynomial relationship between combustion and propulsion characteristics because there was no correlation between them. They stated that although their relationship cannot be used extensively, it can be used as a conditional equation.^50^

## 5 Conclusions

The effect of the addition of BCHMX as a burning modifier to the aluminized HMX/HTPB/AP composite propellant, in which the AP content was partially replaced with these nitramines, was studied (Scheme 1). The substitution was made with pure HMX, a PM, and CACs of HMX/BCHMX, in both cases in a weight ratio of 8:1, in amounts of 10, 20, and 30% of the original AP content. Compared to the physical mixture PM, the used microcrystals CACs have rounded edges (they are a mixture of cocrystals HMX/BCHMX with excess HMX). Investigating the properties of the prepared samples brought the following interesting key findings:

- The incorporation of the physical mixture PM and CACs into the propellant gives it a rubbery character; nevertheless, in the case of CACs with its uniform crystal shape and structure, it is well dispersed and more tightly packed in the propellant with a hardness of 43.05–47.17 °ShA, giving its significantly higher hardness compared to the addition of both pure HMX or PM.
- Compared to the addition of pure HMX with hardness 34.65–37.30 °ShA, the introduction of PM with hardness 32.45–36.56 °ShA into the propellant increases its initiation reactivity significantly more than the addition of CACs.
- The addition of CACs to the propellant increases its burn rate (7.01–8.44 mm/s) and adiabatic flame temperature significantly more than the addition of pure HMX (6.13–7.73 mm/s) or a mechanical mixture PM (6.12–7.81 mm/s).
- The decreasing ignition temperature of the propellant is linearly related to the increase of its burning rate and also heat of combustion (Figure 8b).
- Increasing the hardness of the propellant and reducing its nitramine content leads to an increase in burning rate (Table 6).
- Increasing the CAC content in the propellant decreases the pressure exponent and makes the propellant suitable for use as rocket fuel, whereas pure HMX and the physical mixture (PM) have the opposite effect (Table 7).
- With the exception of the addition of pure HMX, the addition of PM and CACs leads to an inverse relationship between impact sensitivity and burn rate of the respective propellant samples (Figure 8e).
- Substitution of 20 wt % AP by CACs in propellant mass of a given composition significantly increases its hardness and approaches the optimum in terms of overall performance properties (Tables 6 and 7).
- The observed directly proportional relationship between the burn rate and specific impulse indicates both a decrease in impulse with the degree of AP substitution by nitramine (a known effect) (Figure 9) and, as a new finding, a significantly positive effect of CACs on the burn rate, and other propellant performance properties, especially at 20 wt % CACs in the propellant (Tables 6 and 7).

## Supplementary Material



## Notes

(S1) Materials and methods—(S1.1) materials, (S1.2) calculations using REAL software, (S1.3) differential thermal analysis (DTA), (S1.4) powder X-ray diffraction (PXRD), (S1.5) particle size analysis and crystal density specification, (S1.6) FTIR and Raman spectral studies, (S1.7) field emission scanning electron microscope (FESEM), (S1.8) optical microscopy, (S1.9) hardness test, (S1.10) impact sensitivity, (S1.11) friction sensitivity, (S1.12) ignition temperature, (S1.13) vacuum stability test, (S1.14) dynamic mechanical analysis (DMA-Tg), (S1.15) elemental analysis, (S1.16) burn rate measurements, and (S1.17) structural characteristics of the starting EMs (PDF)
