# Supplementary material for: Effective Performance Modifications for a Composite Rocket Propellant via Coagglomerates of Cyclic Nitramines
Source: ACS Omega. 2025 May 27;10(27):29194–208. doi: 10.1021/acsomega.5c01848 (PMC12268416; doi:10.1021/acsomega.5c01848)
Supplement: Supplementary file 1 [file ao5c01848_si_001.pdf]

Supplementary information

Effective Performance Modifications for Composite Rocket Propellant *via* Coagglomerates of Cyclic Nitramines

Veerabhadragouda B. Patil[ψΔ]*, Rafał Lewczuk[Δ], Filip Sazeček[ψ], Paulina Paziewska[Δ],

Petr Stojan[ϕ], Petr Bělina[ŋ], Svatopluk Zeman[ψ]*

[ψ]Institute of Energetic Materials, Faculty of Chemical Technology, University of Pardubice, CZ-532 10 Pardubice, Czech Republic

[Δ]Łukasiewicz Research Network – Institute of Industrial Organic Chemistry, PL-03-236 Warszawa, Poland

[ϕ]OZM Research, CZ-538 62 Hrochův Týnec, Czech Republic

[ŋ]Department of Inorganic Technology, Faculty of Chemical Technology, University of Pardubice, CZ- 532 10 Pardubice, Czech Republic

*Email for correspondence: svatopluk.zeman@upce.cz, iamveerabhadraa@gmail.com

S1 Materials and Methods

S1.1 Materials

HMX and BCHMX used in current work are synthesized in Łukasiewicz Research Network – Institute of Industrial Organic Chemistry; crude HMX was purified by "solvent (dimethyl sulfoxide)/antisolvent (water)" crystallization and the fine crystals were spectrographically identified as α-modification which was used for the samples propellant preparation. The remaining solvents dimethyl sulfoxide and chloroform procured from sigma Andrich. To prepare propellants samples, traditional materials were used as follows (procurements); HTPB (Island Pyrochemical Industries prod.), ammonium per chlorate-AP (Island Pyrochemical Industries prod.), alumina (Benda Lutz prod.) dimeryl di-isocyanate-DDI (Benda Lutz prod.), iron oxide nanoparticles-γ-Fe_2O_3 (Military University of Technology prod.), dioctyl adipate -ADO (Boryszew-Erg prod.), used without further purification. Obtained propellant samples have been analyzed employing specified instrumental techniques.

S1.2 Calculation using REAL software

Prediction of specific impulse, flame temperature, and calculation of oxygen balance was conducted using software REAL,[1] which is a thermodynamic code that is used for computer simulation of chemical equilibrium in complex chemically reacting systems. Enthalpies of formation and the component contens in propellant samples were used as input data (see Table S1).

Table S1. Input characteristics of the propellant components required for calculation

	ΔH_{form} (kJ kg^{-1})	Density (g cm^{-3})	Oxygen balance (%)	Molecular weight
Ammonium Perchlorate	-2517.4	1.95	34.04	117.4891
Iron oxide	-5161.49	5.24	10.02	159.6922
HMX	283.67	1.901	-21.61	296.1551
Physical Mixture-PM (8 HMX:1 BCHMX wt. parts)	363.41	1.881**	-21.10	296.055
CACs (8 HMX:1 BCHMX wt. parts)*	365.13	1.818**	-21.10	296.055
Dioctyl adipate (DOA)	-3278.8	0.925	-263.37	370.5665
Dimeryl diisocyanate (DDI)	-1486.94	0.872	-298.81	588.9905
HTPB	-379.38	0.93	-311.28	140.3973
Aluminum	0.0	2.702	-88.95	26.9815

Note: *) hypothetical formula CAC is $C_{4.01}H_{7.77}N_{8.00}O_{8.00}$

**) experimental value from this paper

S1.3 Differential thermal analysis (DTA)

This analysis was carried out to understand the CACs samples' thermal behaviour was measured using a differential thermal analyzer. A DTA 550 Ex apparatus (OZM Research) was used for thermal analysis of the samples.[2] The measurements were carried out at atmospheric pressure, with the tested sample in direct contact with the air. The tested sample (0.05 g) was placed in a test tube made of Simax glass, 5 mm in diameter and 50 mm long. The reference standard was 0.05 g aluminium oxide. A linear heating rate of 5 ^{o}C min^{-1} was used.

S1.4 Powder X-Ray Diffraction (PXRD)

To understand the morphology and phase purity by PXRD studies and the Scanning electron microscope (SEM) carried out using a nanolayer gold coating(Tuscan, Czech Republic). The PXRD data were collected at room temperature with a Rigaku MiniFlex 600 powder diffractometer with Bragg-Brentano θ-2θ geometry using CuK_{α} radiation (λ = 1.5418 Å, U = 40 kV, I = 15 mA). Data were scanned with an ultrafast detector Dtex ultra over the angular range **2-50° (**2θ) with a step size of **0.02°**.

S1.5 Particle size analysis and crystal density specification

The particle morphology, size distribution, crystal structure and composition analysis of the CACS and the conformers were examined using a MIRA3 LMH scanning electron microscope (Tescan, Czech Republic). Density studies by pycnometer were carried out to evaluate the change in density after conglomeration.

Crystal density was measured using the most reliable technique Gas pycnometer AccuPyc II 1345, this technique is non-destructive as it uses the gas displacement method to measure volume.

S1.6 FTIR and Raman spectral studies

A Nicolet Protege 460 FTIR spectrometer was used to record the IR spectral measurements of the samples using the transmission technique.

Raman spectra were measured by Thermo Scientific™ DXR3 Raman Microscope, employing an excitation laser source (wavelength of 785 nm & power 29 mV), 10x/0.25 objective and using 400 lines/mm grating (3378 to 49 cm^{-1}). For a detailed understanding of the structural aspects of the CACs prepared and the interactions between their components, the FTIR and Raman spectroscopic techniques were used. For FTIR spectroscopy results are summarized in Fig. S2 and for Raman spectroscopy in Fig. S3.

Table S2. Summarized results of FTIR measurements

Assignments	BCHMX	HMX	HMX/BCHMX Phys Mix	HMX/BCHMX CACs
O-N-O-H-- structural bond	3031	3671	--	3054
C-H stretching	3019	3052	3036	3049
Symmetrical N-O stretching	1218	1240	1237, 1261	1210, 1248
Asymmetrical N-O stretching	1562	1539	1527	1534, 1656
C-N stretching [Amino group]	774	733, 763	751, 772	763, 739, 713
C-N stretching [Nitro group]	1326	1315	1347	1369, 1392
Skeletal stretching [Ring]	1137; 1084	1207, 1087	1086, 1138, 1200	1087, 1108, 114, 1210
Symmetric -NO2 Stretching	1273 1209	1270, 1240,	1237, 1261	1248, 1257
Asymmetric -NO2 Stretching	1603, 1554, 1526	1539	1527, 1602	1534, 1603, 1656

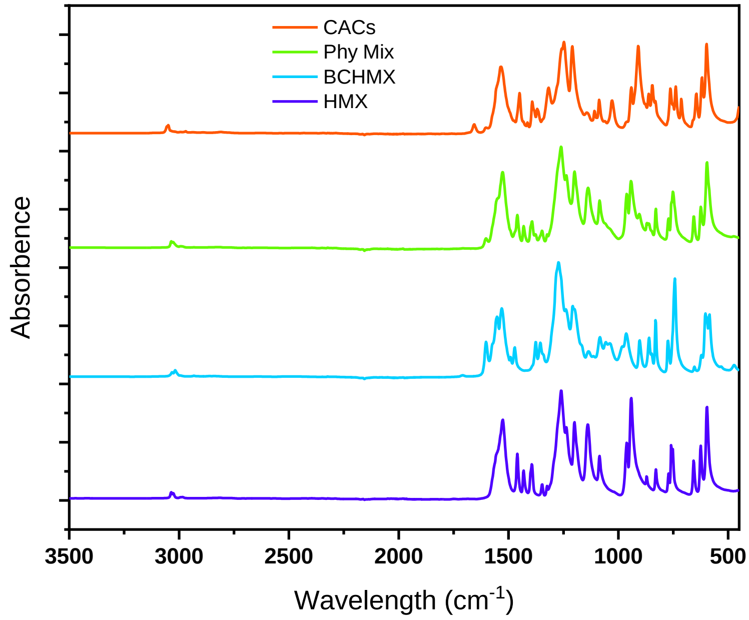

Figure S1. FTIR spectra of Physical mixture, CACs, HMX and BCHMX

Table S3. Summarized results of Raman measurements

Assignments	BCHMX	HMX	HMX/BCHMX Phys Mix	HMX/BCHMX CACs
$–CH_2$ stretching vibration	3020	3037	3037	3037
C–H stretching vibration	2992	2992	2992	2992, 2970
	2932	--	2932	--
Asymmetric $–NO_2$ stretching vibration	1606	--	1606	1607
	1557	1568	1567	1564
-C–H and $–CH_2$ deformation vibration	1299	1315, 1270	1309, 1265, 1248	1320
Symmetric $–NO_2$ stretching vibration	1273, 1264, 1240	1248, 1266	1214, 1265	1217, 1260, 1282
Asymmetric C–H stretching vibration	1194, 1167	1189	1191, 1167	1167
N–N stretching vibration	967, 1058	1079, 951	1058	947
C–C stretching vibration	1058		1083	1089
Ring stretching vibration	967	882	882	878
	906	834	834	929
Ring deformation vibration	850	--	849	848
	906	--	906	878
$–NO_2$ deformation vibration	--	--	--	848
	--	--	--	760
$–NO_2$ Wagging	775	760	774	752
	750	719	760, 750	715

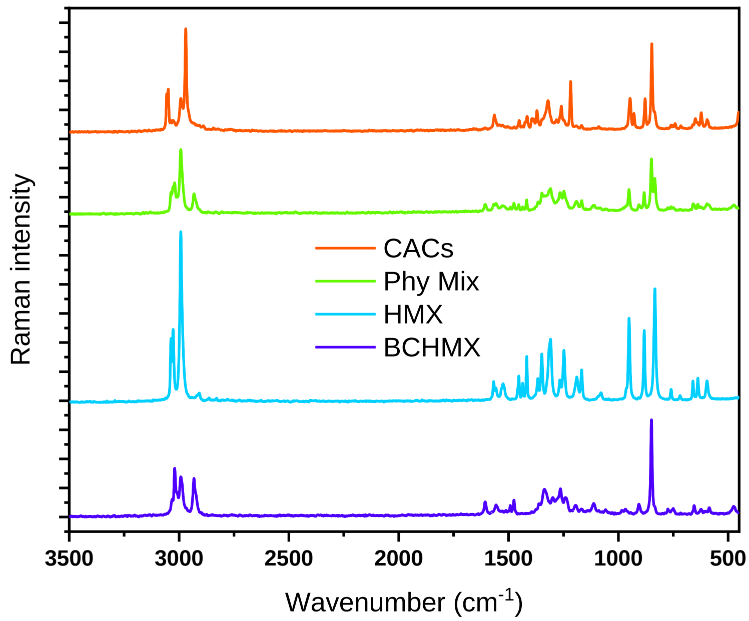

Figure S2. Raman spectra of Physical mixture, CACs, HMX and BCHMX

S1.7 Field emission scanning electron microscope (FESEM)

FESEM analysis carried out using instrument, Jeol JSM-5500-LV (Jeol, Japan), High vacuum, accelerating voltage 10 kV, detected signal – secondary electrons (SEI), working distance – 12-13 mm, spot size – 20, modification of samples – sputtering of gold on their surface, magnification – according to samples properties,

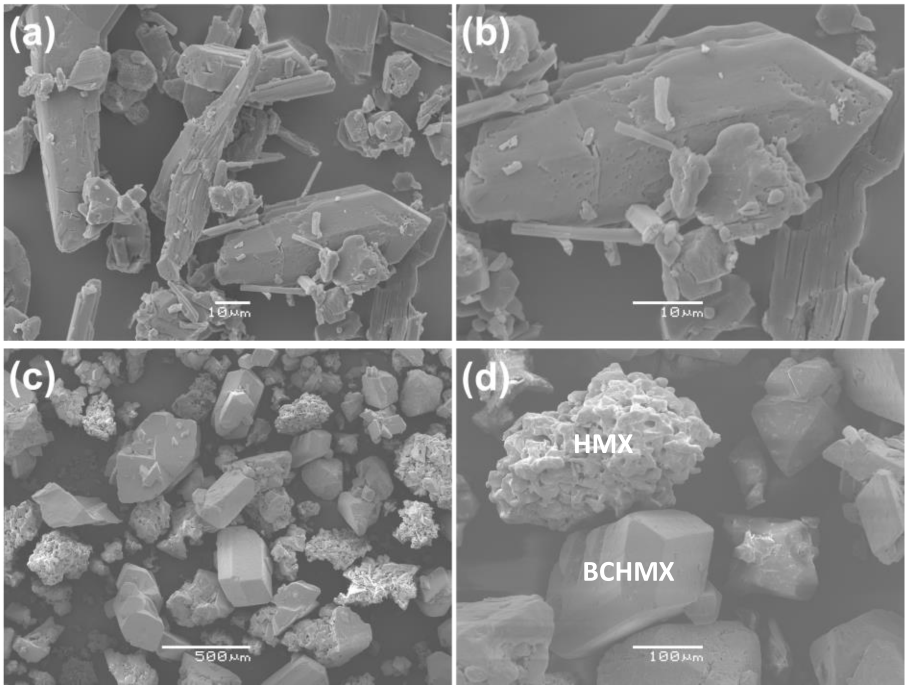

Figure S3. FESEM micrographs a & b) CACs and c & d) PhysMix

S1.8 Optical Microscopy

ZEISS Axiolab 5 optical microscope with EC Epiplan 5x/0.13 M27 objective was used to observe samples of propellants in the reflected light. The images were recorded with Zeiss Axiocam 208 digital camera.

S1.9 Hardness Test

The hardness of tested samples was measured using Shore A hardness tester as per standard ASTM D 2240.[3] The method consists of measurement of the resistance posed by the sample when a needle of specific dimensions and shape is inserted into it. The test result is the average value of sic measurements.

S1.10 Impact Sensitivity

The sensitivity was determined employing a standard impact tester with exchangeable anvil (Julius Peters[4, 5]), BAM BPH series tester make OZM research s. r. o. with the amount of the tested substance being 40 mm^3;[4, 5] the detection was based on sound effect;[5] drop hammers of 5 kg

weight were used.[4, 5] The method European standard PN-EN 13631-4 2003.[5] was utilized to determine the probability levels of the initiation. The obtained sensitivity was expressed as the drop energy, E_{dr}, versus the percentage of initiation. The 50% (E_{dr} 50 %) probabilities of initiation are specified in this report. Both friction and impact sensitivity results are summarized in Table 6 in the main text.

S1.11 Friction Sensitivity

The friction sensitivity of propellants to mechanical stimuli means the ability to undergo an explosive transformation under the influence of mechanical action (e.g., as a result of impact, shot). Friction sensitivity was tested in the Peters apparatus, The method European standard PN-EN 13631-4 2003;[5] in which the force of pressure on two rubbing surfaces is changed (porcelain tile and porcelain stamp, between which the rectangular sample is placed). The pressing force is adjusted by moving the weight into the device. Sensitivity means the highest pressing force with no explosive transformation in six trials.

S1.12 Ignition temperature

This ignition temperature was measured by using the Wood's alloy method,[4a, 4b] as per Polish working standard BN-76/6091-08 (implemented European standard 1363104[5a]) or Czech Public Notice of Czech Mining Authority.[5b] In Polish Inst. Ind. Org. Chem. the apparatus OZM Research DTA 551-Rez was used from the macro-DTA to control the linear rise in the Wood's alloy temperature during the determination of the ignition temperature of the propellant sample. The rate of temperature increase was 5°C min^{-1} and the weight of samples was 100 mg. Measurements were performed in the range of 30-400 °C. In Wood's alloy method, the temperature was measured by a simultaneous heating of three samples of each propellant at temperature increase rate equal to 5 C min^{-1} and visual observation and assessment of propulsion changes. After preheating the alloy to the temperature of 100 °C the test tubes with samples of the propellant (cubes of ca. 40 mg) inserted into it.

S1.13 Vacuum stability test

A modernized STABIL 16-E STABIL VI apparatus was used[7] (manufactured by OZM Research; the original apparatus is described in Ref.[7]) and procedure of measurement according to papers:[2] The amount of the samples used for measurement was 2g. Tests were performed over360 minutes. The temperature for the isothermal measurements was chosen to be 120 °C. The samples in evacuated glass test tubes were placed into the heating block and heated to the desired temperature. Pressure transducers continuously estimated the pressure increase in the glass tubes. The results were in the form of time dependence of the gas pressure evolved per a 1g sample – examples of the corresponding curves up to 300 min measurement are seen papers.[7]

S1.14 Dynamic mechanical analysis (DMA-Tg)

NETZSCH's DMA 242 E Artemis dynamic mechanical analysis (DMA) device was used to determine the mechanical properties of fuel samples. Fuel samples were bent in the dual cantilever mode, measuring 50.0 x 10.0 x 2.0 mm (fixing the samples at two ends). DMA analysis was carried out in an inert gas flow (nitrogen, 50 ml/min) at a temperature range of -120 to 30 °C with a temperature increase rate of 2 °C/min and a frequency of 1 Hz. The deformation has an amplitude of 20 µm. For every fuel sample, there were two measurements taken. The maximum of the loss modulus (E'') peak vs temperature was used to calculate the glass transition temperature. The average of the data is known as the glass transition temperature (T_g).

S1.15 Elemental Analysis

Elemental analysis were performed on Vario MICRO Cube (Elementar) in CHNS mode with calibration on sulphanilamide, using a 1 mg sample for this analysis; results with calculated empirical formulae are represented in Table S4.

Table S4. Elemental analysis of propellant samples

Name	C [%]	H [%]	N [%]	Cl [%]	Empirical Formula
P1	13.87	3.685	10. 50	16.83	$C_{12.31}H_{39.00}N_{8.00}O_{36.77}Cl_{5.06}$
P2	16.02	3.802	13. 24	14.06	$C_{11.28}H_{31.92}N_{8.00}O_{27.87}Cl_{3.36}$
P3	17.22	3.662	16. 12	10.94	$C_{9.97}H_{23.40}N_{8.00}O_{22.61}Cl_{2.15}$
P4	14.14	3.838	10. 17	16.19	$C_{12.97}H_{41.95}N_{8.00}O_{36.26}Cl_{5.08}$
P5	16.02	3.685	14. 27	15.80	$C_{10.47}H_{28.700}N_{8.00}O_{24.64}Cl_{3.51}$
P6	17.61	3.652	15. 12	10,77	$C_{10.87}H_{26.86}N_{8.00}O_{24.49}Cl_{2.25}$
P7	14.05	3.713	10. 67	16.19	$C_{12.28}H_{38.67}N_{8.00}O_{36.34}Cl_{4.81}$
P8	15.53	3.615	13. 12	13.74	$C_{11.05}H_{30.65}N_{8.00}O_{28.84}Cl_{3.31}$
P9	18.50	3.628	15. 50	10.42	$C_{11.14}H_{26.00}N_{8.00}O_{23.47}Cl_{2.08}$

Note: Values of elemental analysis are averaged values of four trials for each sample

S1.16 Burn rate measurements

The burning rate of rocket propellant is one of its basic characteristics. The rate of burning depends on the pressure. The linear velocity of burning versus the pressure was established in the laboratory rocket motor (LRM). The charges prepared for testing had a form of rectangular cylinders with dimensions 30×14.8×8 (length×outer diameter×inner diameter) mm. Calculation of linear velocity of burning was made according to a methodology detailed in paper.[9] The critical diameter of the nozzle was 2.6, 2.8, and 3.0 mm for measurements.

S1.17 Structural characteristics of the starting EMs

To better understand the mutual interaction of component molecules in CACs, their spectral data are compared in the main text with characteristics of molecular crystals of these components taken from the CCDC crystal database. The ORTEP views of the used nitramines, HMX and BCHMX, serving as references for the main text, are summarized below in their figure forms (Figures S4 & S5):

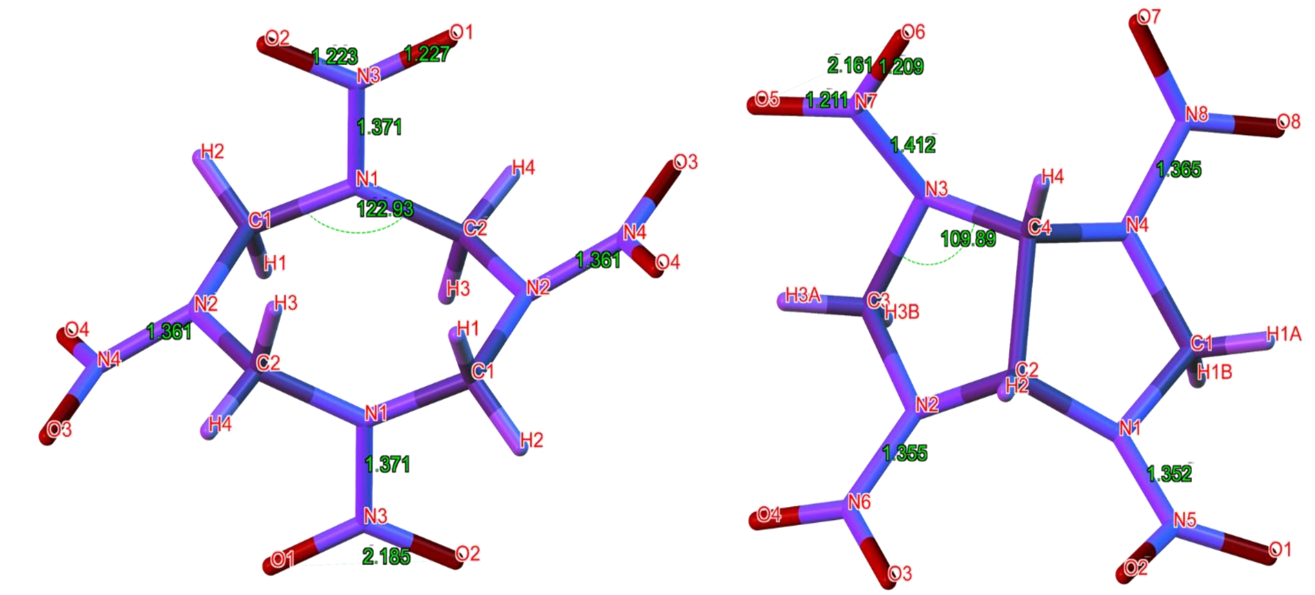

a) β-HMX with inter-atomic distances (pm)[10]　　b) BCHMX with inter-atomic distances (pm)[11]

Figure S4. 3D molecular structures of β-HMX and BCHMX

References

[1] G.V. Belov, REAL for Windows computer modeling of complex chemical equilibrium at high pressures and temperature, *Software, version 3.0*, Moscow State Technical University, Moscow, **2001.**

[2] M. Krupka, Devices and equipments for testing of energetic materials, *Proc. 4th Int. Seminar NTREM*, Univ. Pardubice, **2001**, p. 222.

[3] *Standard Test Method for Rubber Property* - Durometer Hardness. ASTM D2240-05, Annual Book of ASTM Standards, **200**5.

[4] a) M. Suceska, Test Method for Explosives, Springer-Verlag, New York, **199**5, pp. 11-16.
b) Anonymous, Explosives, thermal sensitiveness and explosiveness tests, *STANAG 4491* - Ed: 3rd, Eur. Def. Agency, March 21st, **2022**.

[5] a) Explosives for civil uses high explosives Part 4: Determination of sensitivity to impact; *The European standard* 1363104, **2002**.

b) Public Notice of Czech Mining Authority No. 246/1996 *Collection of Czech Laws*, Establishing more Detailed Conditions for Allowing Explosives, Explosive Objects and Aids into Use, and Their Testing, Aug. 13th, **1996**, pp. 3200-3208.

[6] B. Florczak, R. Bogusz, W. Skupiński, M. Chmielarek, Study of the effect of nitrated hydroxy-terminated polybutadiene (NHTPB) content on the properties of heterogeneous rocket propellant, *Cent. Eur. J. Energ. Mater*. **2015,** 12 (4), 841-854.

[7] V. Kučera, B. Vetlický, Investigation of the decomposition processes in single-base propellants under vacuum using minicomputer-controlled automated apparatus, *Propellants Explos. Pyrotech*. **1985,** 10, 65-70.

[8] S. Zeman, A. Elbeih, A. Hussein, T. Elshenawy, M. Jungova, Q.-L. Yan, A modified vacuum stability test in the study of initiation reactivity of nitramine explosives, *Thermochim. Acta* **2017,** 656, 16–24.

[9] P. Stojan, The use of low pressure clossed vessel and rocket motor for measurements of burning rate of rocket solid propellants, *Proc. 9th Int. Seminar NTREM*, Univ. Pardubice, **2006**, pp. 730-735.

[10] L. Zhang et al., Experimental Study of the Crystal Habit of High Explosive Octahydro-1,3,5,7-tetranitro-1,3,5,7-tetrazocine (HMX) in Acetone and Dimethyl Sulfoxide, *Cryst. Growth Des*. **2020,** 20(10), 6622–6628.

[11] D. Klasovitý, S. Zeman, A. Růžička, M. Jungová, M. Roháč, cis-1,3,4,6-Tetranitrooctahydroimidazo-[4,5-d]imidazole (BCHMX), its properties and initiation reactivity, *J. Hazard. Mater*. **2009,** 164(2–3), 954–961.
